# Commentary: Extracellular peptidase hunting for improvement of protein production in plant cells and roots

**DOI:** 10.3389/fpls.2015.00557

**Published:** 2015-07-21

**Authors:** Karl J. Kunert, Priyen Pillay

**Affiliations:** Molecular Plant Physiology Group, Plant Science Department, Forestry and Agricultural Biotechnology Institute, University of PretoriaPretoria, South Africa

**Keywords:** proteases, recombinant protein production, protein stability, *in silico* analysis, molecular pharming

Despite much recent success in plant-based protein production, key challenges, such as undesired plant proteolytic activities, still severely compromises current recombinant protein production with peptidases affecting protein stability (Pillay et al., 2013). The paper by Lallemand et al. (2015) reporting about identification of extracellular peptidases compromising protein production in plant cells and roots is therefore an excellent contribution to ultimately advance our understanding of peptidase action in plant-based recombinant protein production (Lallemand et al., 2015). Since research has so far not paid a great amount of attention to this problem, a more detailed view, as taken in the paper, is highly beneficial to elucidate such peptidases in the extracellular space. This offers great benefits in terms of protein stability and higher protein production yield.

Previous approaches used to address this challenge in plants has for example included peptidase silencing by applying RNA interference technology (Voinnet et al., 2003; Hatsugai et al., 2004) and also co-expressing specific protease inhibitors as “companions” to limit specific protease activities (Goulet et al., 2010, 2012; Pillay et al., 2012). However, silencing a specific peptidase or co-expressing a “companion” protease inhibitor always bears the risk of vital plant metabolic pathways also being affected (Van der Vyver et al., 2003; Senthil-Kumar et al., 2007). This can compromise efficient recombinant protein production in a plant-based system. In addition, work on Arabidopsis, as already done by Lallemand et al. (2015), with its existing wealth of transcriptome and gene data (The_Arabidopsis_Genome_Initiative, 2000) will enable future identification of similar peptidases in other plant species when comparative genomics approaches are applied in combination with Next Generation Sequencing.

By investigating two plant species (*Arabidopsis thaliana* and *Nicotiana tabacum*); the Lallemand et al. (2015) study particularly unraveled that root-secretion production contained more peptidase activity than, for example, the extracellular medium of cell suspensions. A less proteolytic enriched environment is certainly more favorable for the production of recombinant proteins, especially antibodies. This key finding has, therefore, not only significantly extended our understanding how particular plant species contribute to proteolytic activity and type of peptidase produced but has also contributed to advancing our understanding on how proteases in different plant parts can compromise recombinant protein stability. The study has whereby set a strong working basis for exploring, in the future, proteolytic action in greater depth.

Lallemand et al. (2015) also focused on establishing geno-transcriptome data. By also tapping into the wealth of existing peptidase data, Lallemand et al. (2015) further carried out an in-depth *in silico* analysis of existing Arabidopsis genome and transcriptome data. Remarkably, the search resulted in identification of serine and metallo-peptidases as main peptidases involved in proteolytic processes. These peptidases were consistently expressed in the two investigated production systems. By applying the approach of merging activity assays with geno-transcriptome data, specific Ser-peptidases, potentially responsible for target degradations, were identified. Lallemand et al. (2015) proposed that these peptidases should first be prime candidates for modification to improve protein stability.

Specific inhibition of Ser-proteases is certainly an attractive idea which is also supported by previous findings (Goulet et al., 2012). However, the question still remains, how many other proteases are there particularly in plants currently applied in recombinant protein production and what role(s) do they play in protein production and stability. For example commercial companies are primarily using *Nicotiana benthamiana* and also the unconventional method of producing proteins in carrot cells is applied. These plant species might have very different protease profiles. Investigating such systems for protein production from a plant-based perspective, suggests commercial preferences in industry which are excellent indicators for researchers to adopt in their methodology. Consequently, more definitive investigations are required in protease profiling with the option to avoid plant species with a specific profile unfavorable for the production of a specific recombinant protein. In this regard, recent Next generation sequencing and also proteomics approaches for protease profiling (Vandenabeele et al., 2003; van Wyk et al., 2014) will allow the identification of a great number of peptidases as well as the establishment of their particular expression profiles in plant species targeted for recombinant protein production. In addition, more focused assessments in recombinant protein susceptibility to proteases have to be carried out to identify potential cleavage sites within the protein. These considerations and risks are encapsulated in our pipeline for enhancing protein expression (Figure 1) which illustrates two stages where proteins are most vulnerable to proteolysis. A different complement of plant-derived proteases may be released during the extraction process from a cellular compartment that is different to that where the target protein is originally localized and thus may also co-purified during the purification process. Once the inherent susceptibility of the target protein is determined, appropriate inhibitors can be used to ameliorate the negative effects of proteases during extraction and purification.

**Figure 1:**
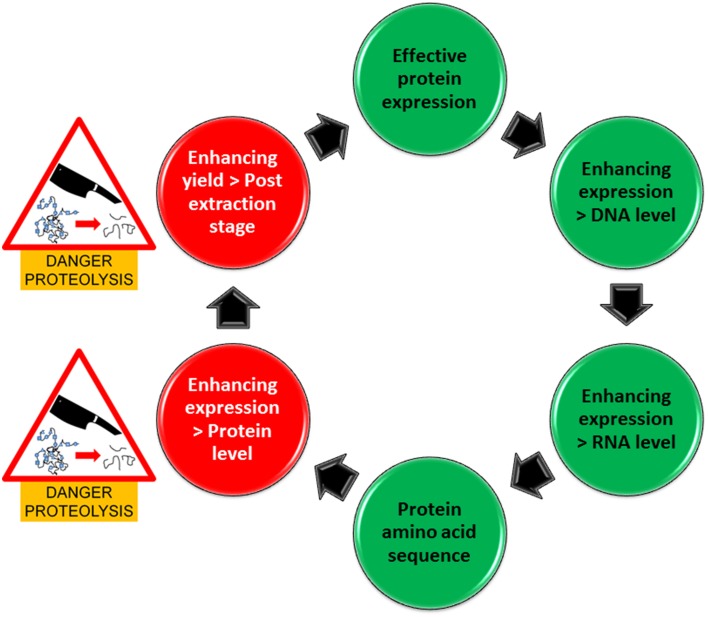
**Pipeline for enhancing protein production in expression systems**. Green circles represent areas that are free of danger from proteolysis whilst red circles represent areas where there is a danger of proteolysis.

Without doubt, the study is, as Lallemand et al. (2015) have already outlined, an excellent starting point to develop new strategies for identifying proteolytic activity with the goal of enhancing recombinant protein stability.

## Funding

This work was supported by National Research Foundation (NRF) as NRF incentive funding to KK and a NRF bursary to PP.

### Conflict of interest statement

The authors declare that the research was conducted in the absence of any commercial or financial relationships that could be construed as a potential conflict of interest.
